# Chronic Rho-kinase inhibition improves left ventricular contractile dysfunction in early type-1 diabetes by increasing myosin cross-bridge extension

**DOI:** 10.1186/s12933-015-0256-6

**Published:** 2015-07-22

**Authors:** Mark T Waddingham, Amanda J Edgley, Alberto Astolfo, Tadakatsu Inagaki, Yutaka Fujii, Cheng-Kun Du, Dong-Yun Zhan, Hirotsugu Tsuchimochi, Naoto Yagi, Darren J Kelly, Mikiyasu Shirai, James T Pearson

**Affiliations:** Department of Medicine, St. Vincent’s Hospital, University of Melbourne, Melbourne, VIC Australia; Department of Physiology, Monash University, Clayton, VIC Australia; Australian Synchrotron, Clayton, VIC Australia; Department of Medical Physics and Bioengineering, University College of London, London, England, UK; Department of Cardiac Physiology, National Cerebral and Cardiovascular Center Research Institute, Suita, Osaka Japan; Japan Synchrotron Radiation Research Institute, Harima, Hyogo Japan; Monash Biomedical Imaging Facility, Monash University, Clayton, VIC Australia

**Keywords:** Diabetes, Diabetic cardiomyopathy, Myocardium, Sarcomere, Rho-kinase, Small angle X-ray scattering

## Abstract

**Background:**

Impaired actin–myosin cross-bridge (CB) dynamics correlate with impaired left ventricular (LV) function in early diabetic cardiomyopathy (DCM). Elevated expression and activity of Rho kinase (ROCK) contributes to the development of DCM. ROCK targets several sarcomeric proteins including myosin light chain 2, myosin binding protein-C (MyBP-C), troponin I (TnI) and troponin T that all have important roles in regulating CB dynamics and contractility of the myocardium. Our aim was to examine if chronic ROCK inhibition prevents impaired CB dynamics and LV dysfunction in a rat model of early diabetes, and whether these changes are associated with changes in myofilament phosphorylation state.

**Methods:**

Seven days post-diabetes induction (65 mg/kg ip, streptozotocin), diabetic rats received the ROCK inhibitor, fasudil (10 mg/kg/day ip) or vehicle for 14 days. Rats underwent cardiac catheterization to assess LV function simultaneous with X-ray diffraction using synchrotron radiation to assess in situ CB dynamics.

**Results:**

Compared to controls, diabetic rats developed mild systolic and diastolic dysfunction, which was attenuated by fasudil. End-diastolic and systolic myosin proximity to actin filaments were significantly reduced in diabetic rats (P < 0.05). In all rats there was an inverse correlation between ROCK1 expression and the extension of myosin CB in diastole, with the lowest ROCK expression in control and fasudil-treated diabetic rats. In diabetic and fasudil-treated diabetic rats changes in relative phosphorylation of TnI and MyBP-C were not significant from controls.

**Conclusions:**

Our results demonstrate a clear role for ROCK in the development of LV dysfunction and impaired CB dynamics in early DCM.

**Electronic supplementary material:**

The online version of this article (doi:10.1186/s12933-015-0256-6) contains supplementary material, which is available to authorized users.

## Background

Diabetes mellitus is a rapidly escalating global epidemic with the International Diabetes Federation predicting that the global incidence of diabetes will rise to 552 million people by 2030 [1]. The most common complication of diabetes is cardiovascular disease and, is a leading cause of morbidity and mortality in patients [2]. More specifically, diabetes is associated with impaired myocardial function, independent of coronary vascular disease and hypertension, and termed diabetic cardiomyopathy (DCM) [3]. In general, DCM has been recognized by impaired left ventricular (LV) isovolumetric (active) and passive relaxation, myocardial fibrosis and cardiomyocyte hypertrophy [4]. However, four decades of extensive research on experimental animal models and significant advances in cardiac clinical imaging now suggests that DCM is a progressive disease, with LV contractile dysfunction beginning early in the time course of diabetes, ahead of structural remodeling [5]. Subcellular alterations to the cardiomyocyte such as diminished Ca^2+^ handling ability, reduced ATPase activity and sarcomeric dysfunction might all contribute to LV contractile dysfunction in early diabetes [6, 7].

Utilizing synchrotron radiation as a source for small angle X-ray scattering (SAXS), we have recently reported impaired actin–myosin cross-bridge (CB) dynamics in the in situ beating heart, 3 weeks post streptozotocin (STZ)-induced diabetes in rats [8]. We demonstrated that in the hearts of diabetic rats, myosin heads are displaced away from the actin thin-filament during diastole, leading to suppressed systolic myosin transfer to actin, and an assumed reduction in strong CB formation as the rate of pressure development was decreased [8]. Given that myosin interfilament spacing did not differ between the groups, we speculated that LV contractile dysfunction driven by impaired CB dynamics in early diabetes might be attributed to a reduction in the activity of myosin accessory proteins, myosin light chain-2 (MLC-2) or cardiac myosin binding protein-C (MyBP-C), which meticulously regulate myosin head extension on a beat-to-beat basis [9–13].

The Rho kinases (ROCK), ROCK1 and ROCK2 are Rho-associated kinases activated by the small GTP-binding protein RhoA. The RhoA/ROCK pathway is involved in a diverse range of cellular processes in the cardiovascular system, although of particular interest is the vital role that ROCK plays in regulating CB dynamics in vascular smooth muscle and cardiac muscle cells [14]. ROCK has been widely implicated in various animal models of diabetic [15, 16] and non-diabetic diastolic LV dysfunction [17–19]. Importantly, a recent clinical study highlighted the beneficial effects of short-term fasudil (a specific ROCK inhibitor) therapy on improving both active (isovolumetric) and passive myocardial relaxation in diabetic patients [20]. Others have reported that ROCK directly phosphorylates several thick and thin filament contractile proteins including MyBP-C, MLC-2, troponin I (TnI) and troponin T (TnT), and might be involved in myofilament dysfunction [21]. This may in part, explain improved active diastolic function in diabetic patients treated with fasudil for 2 weeks [20].

A prolonged rate of active relaxation is an established feature of early DCM, even as early as 2–3 weeks post STZ diabetes induction in rats [22, 23]. Reduced myosin head extension is correlated with impaired LV active relaxation [8] and we have shown elevated myocardial ROCK expression with diabetic coronary dysfunction [24]. Therefore, we examined if ROCK inhibition with fasudil restored CB dynamics and subsequently LV function in the STZ rat model at an early stage of DCM, and whether these changes are associated with changes in ROCK expression and myofilament phosphorylation state in isolated cardiac tissues.

## Methods

### Animals

Reporting of animal studies in this paper comply with the ARRIVE guidelines for animal research [25]. All animal experiments were approved by the local Animal Ethics Committees and conducted in accordance with the National Health and Medical Research Council of Australia Code of Conduct for the Care of Animals for Scientific Purposes and the guidelines of the Physiological Society of Japan. Two independent cohorts of male Sprague–Dawley rats (7–8 weeks old) were sourced from Animal Resources Centre (Perth, WA, Australia) for global cardiac function and protein phosphorylation studies (Experiment 1) or Japan SLC (Kyoto, Japan) for synchrotron small angle X-ray scattering (SAXS) (Experiment 2). Both cohorts of rats randomly received either a single vehicle injection of sodium citrate buffer (0.1 M, pH 4.5) (control) or streptozotocin (STZ; 65 mg/kg ip) to induce type 1 diabetes. Seven days later, STZ-injected rats were randomized to receive daily subcutaneous injections of the ROCK inhibitor, fasudil (10 mg/kg/day; LC Laboratories, MA, USA) or the equivalent volume of the saline vehicle (1 ml/kg) and followed for a further 14 days. Control rats received only saline vehicle injections, except in experiment 2, we also included a fasudil-treated control group to examine if tonic ROCK activation plays a role in CB disposition regulation in the non-diabetic heart. Body weight and blood glucose concentrations were measured weekly in both cohorts. Only rats with a blood glucose concentration >15 mmol/L were considered diabetic. All rats were housed in a controlled environment and given access to food and water ad libitum.

### Surgical preparation of animals

Under deep surgical anesthesia (pentobarbital sodium; 60 mg/kg, ip), confirmed by the complete absence of the pedal reflex, rats were intubated and artificially ventilated (~40% O_2_; 10 ml/kg). Rats were constantly monitored as previously described [26] and supplemental doses of pentobarbital were administered throughout the experiments to maintain adequate anesthesia. In experiment 2, pancuronium bromide was also used in addition after confirming an adequate depth of anesthesia to eliminate spontaneous breathing while the ventilator was briefly switched off during recordings [26]. Body temperature was maintained at 37°C throughout the experimental protocol with the use of a rectal thermistor coupled with a thermostatically controlled heating pad.

#### Experiment 1

Briefly, the right carotid artery was isolated and catheterized with a 2Fr pressure–volume (PV) conductance catheter (SPR-838, Millar Instruments, TX, USA) and advanced retrograde into the LV. The left jugular vein was also cannulated for fluid replacement and hypertonic saline bolus infusion. Subsequently, the abdomen was opened and loose ligatures placed around the inferior vena cava (IVC) and portal vein to facilitate pre-load reduction [27].

#### Experiment 2

For synchrotron SAXS studies, rats underwent a thoracotomy to allow an unobstructed path to the heart. A continuous drip-flow of lactate Ringers solution was used to prevent the drying of the exposed heart and lungs. A PV catheter was advanced into the LV as described above to allow simultaneous recording of SAXS and PV data [8]. The right jugular vein was then cannulated for fluid replacement and to maintain a stable blood and LV volume. The right femoral artery was also cannulated for the continual monitoring of blood pressure. The heart was then partly restrained with a plastic receptacle inserted beneath the posterior apex to ensure maintenance of myocardial depth during imaging experiments [26, 28].

### Experiment 1 protocol

Load-dependent and load-independent measures of cardiac function were assessed by PV loop analysis [29–31]. Continuous PV loop monitoring was performed throughout the experiments however, for PV loop recordings, the ventilator was switched off briefly (~5 s) with the rat apneic to reduce breathing motion artifacts. PV data were acquired under steady state conditions and during preload reduction by the occlusion of the IVC and portal vein by methods previously described [30, 31]. The volume signal was calibrated by the validated hypertonic saline method [32]. PV data were recorded using CHART (v5.5.6, AD Instruments, NSW, Australia) and subsequently analyzed offline using PVAN (v3.5, Millar Instruments).

### Experiment 2 SAXS system and protocol

#### X-ray source, camera and diffraction recordings

Experiments were conducted at Beamline 40XU at the Japanese Synchrotron Radiation Research Institute (SPring-8), Hyogo, Japan. In brief, the myocardial surface was aligned at an oblique tangent to a collimated quasi-monochromatic X-ray beam with the dimensions, wavelength and flux as previously described [8, 26, 28] with the rat approximately 3 m away from the detector [8, 28]. SAXS patterns (<2.1 s) were acquired at 15 ms intervals using an image intensifier (V5445P, Hamamatsu Photonics, Hamamatsu, Japan) and a fast charge-coupled device camera (C4880-80-24A, Hamamatsu Photonics). Diffraction patterns were recorded using HiPic32 acquisition software (v5.1.0, Hamamatsu Photonics).

#### Protocol

SAXS recordings were obtained during an intravenous infusion of lactate (4 ml/h; lactate Ringers solution, Otsuka Pharmaceuticals, Osaka, Japan). Rats were then killed with a potassium chloride (KCl) bolus (0.1 M) to arrest the heart in diastole. SAXS patterns were also recorded post-KCl administration during muscle quiescence.

### X-ray diffraction pattern analysis

SAXS patterns were analyzed using an in-house designed software (X-RAT) utilizing commercial visualization tools and algorithms (IDL8.2, Exelis, VA, USA). Myofilament lattice spacing calibration was made using the 63.5 nm meridional reflection from a dried chicken tendon at the beginning of experiments. SAXS sequences were loaded into X-RAT and the arc radians of the 1,0 and 1,1 equatorial reflections specified from the image center. A manual second order polynomial curve fitting was performed within user defined inner and outer limits. The integrated intensity of the 1,0 and 1,1 reflection intensities were then determined by the areas under the reflection peaks, defined as I_1,0_ and I_1,1_, respectively. The integrated intensity of the 1,1 reflection was then corrected by a √3 multiplication as previously discussed by Jenkins et al. [8] prior to analysis. As the number of fibers in the beam path changes during contraction the equatorial intensity ratio (I_1,0_/I_1,1_) was used to determine the transfer of myosin mass, presumed to be CBs, from the thick to the thin filament. The 1,0 reflection lattice spacing (d_1,0_) was obtained from the center of gravity of the integrated 1,0 reflection at end diastole and maximum systolic spacing.

Radial transfer of myosin heads to actin was calculated as an absolute measure of myosin mass transfer based on the methods previously described [8]. Briefly, intensity ratios during complete muscle quiescence (post KCl administration) and the minimum attainable intensity ratio of 0.2 during rigor state (maximum actin binding site occupancy) in both control and diabetic hearts were obtained. Linear regression was then performed to interpolate the peak systolic and end-diastolic myosin mass transfer to actin (% of maximum in rigor state) [8].

### Tissue collection

At the conclusion of all experiments, hearts were rapidly excised and sliced transversely. The apex portion minced and snap-frozen in liquid nitrogen and stored at −80°C and the remainder was fixed in 4% paraformaldehyde.

### Histology

Sections of 4 μm thickness were stained with hematoxylin and eosin (H&E) to assess cardiomyocyte cross-sectional area in approximately 30 single nucleated cells in each section. Picrosirius red was used to investigate LV interstitial fibrosis. All histological sections were imaged using the Aperio ScanScope XT Slide Scanner (Aperio Technologies, CA, USA). The proportional area of positive staining was quantified using the Positive Pixel Count algorithm (v9.1) on Aperio ImageScope software.

### Tissue preparation for SDS–PAGE and Western blotting

At least 100 mg of LV tissues were homogenised in ice-cold lysis buffer containing protease and phosphatase inhibitors. The protein concentration of each sample was determined using a Bradford protein assay.

### Myofilament protein phosphorylation

Approximately 10 μg of protein was loaded per sample. Proteins were then subjected to SDS–PAGE using Bio-Rad AnykD ™ (Bio-Rad, CA, USA) gradient mini-gels. Gels were then fixed, washed and stained with a specific phosphoprotein stain, Pro-Q Diamond (Invitrogen, OR, USA) according to the manufacturers protocol and imaged using a ChemiDoc MP imager system (Bio-Rad). After imaging, gels were then post-stained with SYPRO Ruby (Invitrogen) overnight for total protein. Relative phosphorylation of myofilament proteins were determined by dividing the Pro-Q Diamond signal by the SYPRO Ruby total protein signal [33].

### Western blotting

For western blotting, 30–75 μg of protein was loaded on 7.5 or 12% polyacrylamide mini-gels, subjected to SDS–PAGE and subsequently transferred onto PVDF membranes. After blocking with 5% non-fat milk, membranes were incubated in primary antibodies against ROCK1 (1:500), ROCK2 (1:200) (Abcam, Cambridge, UK), pRhoA^Ser188^ (1:1,000) and RhoA (1:1,000) (SantaCruz Biotechnology, CA, USA) at 4°C overnight [34]. Following incubation in an anti-rabbit HRP conjugated secondary antibody (Dako, Glostrup, Denmark; 1:2,500) for 1 h at room temperature, proteins were detected using the ECL detection method. Membranes were then re-probed with β-actin (Abcam, 1:1,000) or GAPDH (Abcam, 1:10,000), which served as a loading control. Bands were analysed by densitometry using Image Lab Software (Bio-Rad).

### Statistical analysis

All data is expressed as mean ± SEM unless otherwise stated. Differences between groups were assessed by a one-way ANOVA with a Fischer’s least significant difference post hoc test. Linear regression was performed to test for significant correlations. All statistical analysis were performed using GraphPad Prism (v6.0, GraphPad, CA, USA) with P < 0.05 considered statistically significant.

## Results

### General animal characteristics

The administration of STZ to rats in both experimental cohorts resulted in a significant three to four fold increase in fasting blood glucose concentrations and a significantly lower body weight compared to control rats (Table 1; Additional file 1: Table S1). Fasudil treatment in control and diabetic rats did not affect blood glucose concentration or body weight. In experiments 1 and 2, LV weights tended to be lower in diabetic rats compared to their control counterparts. When normalized to body weight (LV:BW ratio) however, no significant difference was observed between all groups in either experimental cohort (Table 1; Additional file 1: Table 1). Similarly, mean arterial pressure of rats did not significantly differ amongst the groups in either experimental cohort (Table 1; Additional file 1: Table S1).

**Table 1:** General characteristics of rats from experiment 2

	Control	Control + fasudil	Diabetic	Diabetic + fasudil
*N*	5	4	7	7
Body weight (g)	381 ± 6	384 ± 17	300 ± 20**	275 ± 17^#^
Fasted blood glucose (mmol/L)	7.3 ± 0.3	7.5 ± 0.1	28.5 ± 1.8^##^	32.3 ± 0.7^##^
Mean arterial pressure (mmHg)	95.8 ± 5.5	105.3 ± 9.2	83.9 ± 7.3	94.4 ± 10.9
Left ventricle weight (g)	0.76 ± 0.02	0.74 ± 0.03	0.66 ± 0.06	0.59 ± 0.04^**#**^
Left ventricle weight:body weight (mg/g)	2.0 ± 0.08	1.9 ± 0.04	2.3 ± 0.04	2.2 ± 0.13

Data expressed as mean ± SEM.

* P < 0.05, ** P < 0.01 and ^##^P < 0.0001 vs. control rats.

### Myocardial structure

Cardiomyocyte cross-sectional area did not significantly differ across all groups in rats from either experiment, indicating that cardiac hypertrophy was not found in the diabetic groups (Additional file 1: Figure S1, shown for experiment 2) Similarly, LV interstitial collagen ratio (fibrosis scores) were similar among the groups in both experimental cohorts (Additional file 1: Figure S2).

### Global left ventricular function: experiment 1

In comparison to control rats, diabetic rats exhibited a ~17% reduction in heart rate (350 ± 12 vs. 290 ± 12, P < 0.01, Table 2). Diabetic rats treated with fasudil maintained a significantly lower heart rate to that of control rats (350 ± 12 vs. 299 ± 13, P < 0.01, Table 2). Three weeks of STZ-induced diabetes resulted in mild systolic dysfunction compared to controls, evident as a suppressed LV end systolic pressure (127 ± 6 vs. 95 ± 9 mmHg, P < 0.01, Table 2), a significant 35% reduction in preload recruitable stroke work (PRSW; 74 ± 10 vs. 48 ± 4 mmHg, P < 0.05, Table 3) in addition to non-significant 46% and 35% reductions in the end-systolic pressure volume relationship (ESPVR) and the relationship of the rate of pressure development and ED volume (dP/dt-EDV), respectively (Table 3). Impairment in LV active relaxation was also found in diabetic rats evidenced by a prolonged mean rate of LV pressure decay (dP/dt_minimum_, P < 0.05, Table 2) and increased mean Tau relaxation time (12 ± 0.3 vs. 14.4 ± 0.7 ms, P < 0.05, Table 2) when compared to controls. Passive compliance of the diabetic heart did not differ from that of controls as demonstrated by similar LV ED pressures (7.5 ± 0.9 vs. 7.8 ± 1 mmHg, NS, Table 1) and ED pressure volume relationships (EDPVR; 0.03 ± 0.005 vs. 0.02 ± 0.001 mmHg/μl, NS, Table 3).

**Table 2:** Load dependent indices of left ventricular function as measured by pressure–volumetry from Experiment 1

	Control	Diabetic	Diabetic + fasudil
Heart rate (BPM)	350 ± 12	290 ± 12**	299 ± 13**
End systolic pressure (mmHg)	127 ± 6	95 ± 9**	115 ± 5
End diastolic pressure (mmHg)	7.5 ± 0.9	7.8 ± 1	8.7 ± 1
Stroke volume (μl)	168 ± 20	184 ± 8	193 ± 17
Cardiac output (ml/min)	59.4 ± 8.1	53.5 ± 3.5	57.7 ± 5.6
Ejection fraction (%)	66 ± 8	60 ± 7	50 ± 3
dP/dt_maximum_ (mmHg/s)	7,391 ± 602	6,616 ± 470	7,096 ± 641
dP/dt_minimum_ (mmHg/s)	−8,210 ± 632	−5,917 ± 502*	−6,439 ± 569*
Tau Glantz (ms)	12 ± 0.3	14.4 ± 0.7*	14.2 ± 0.8*

Data expressed as mean ± SEM.

dP/dt_maximum_, maximum rate of left ventricular pressure development; dP/dt_minimum_, minimum rate of left ventricular pressure decay.

* P < 0.05 and ** P < 0.01 vs. control rats.

**Table 3:** Load-independent cardiac indices as measured by left ventricular pressure-volumetry from Experiment 1

	Control	Diabetic	Diabetic + fasudil
ESPVR (mmHg/μl)	0.52 ± 0.15	0.28 ± 0.05	0.33 ± 0.01
PRSW (mmHg)	74 ± 10	48 ± 4*	63 ± 6
EDPVR (mmHg/μl)	0.03 ± 0.005	0.02 ± 0.001	0.03 ± 0.005
dP/dt-EDV (mmHg/s/μl)	23 ± 4	15 ± 1	22 ± 3

Data expressed as mean ± SEM.

ESPVR, end systolic pressure volume relationship; PRSW, preload recruitable stroke work; EDPVR, end diastolic pressure volume relationship; dP/dt-EDV, relationship of rate of pressure development and end diastolic volume.

* P < 0.05 vs. control.

The systolic dysfunction associated with diabetes was alleviated in diabetic rats treated with fasudil, as no significant difference in end systolic pressure or PRSW (Tables 2, 3) was found when compared to control rats. In contrast, impairment in active diastolic relaxation associated with diabetes was not significantly improved by fasudil. Although there was a ~10% increase in the rate of pressure decay in diabetic rats treated with fasudil in comparison to vehicle treated diabetic rats, LV pressure decay rate was significantly prolonged when compared with controls (dP/dt_minimum_, Table 2).

### Rho kinase expression and myofilament protein phosphorylation in the left ventricle: experiment 1

**Figure 1:**
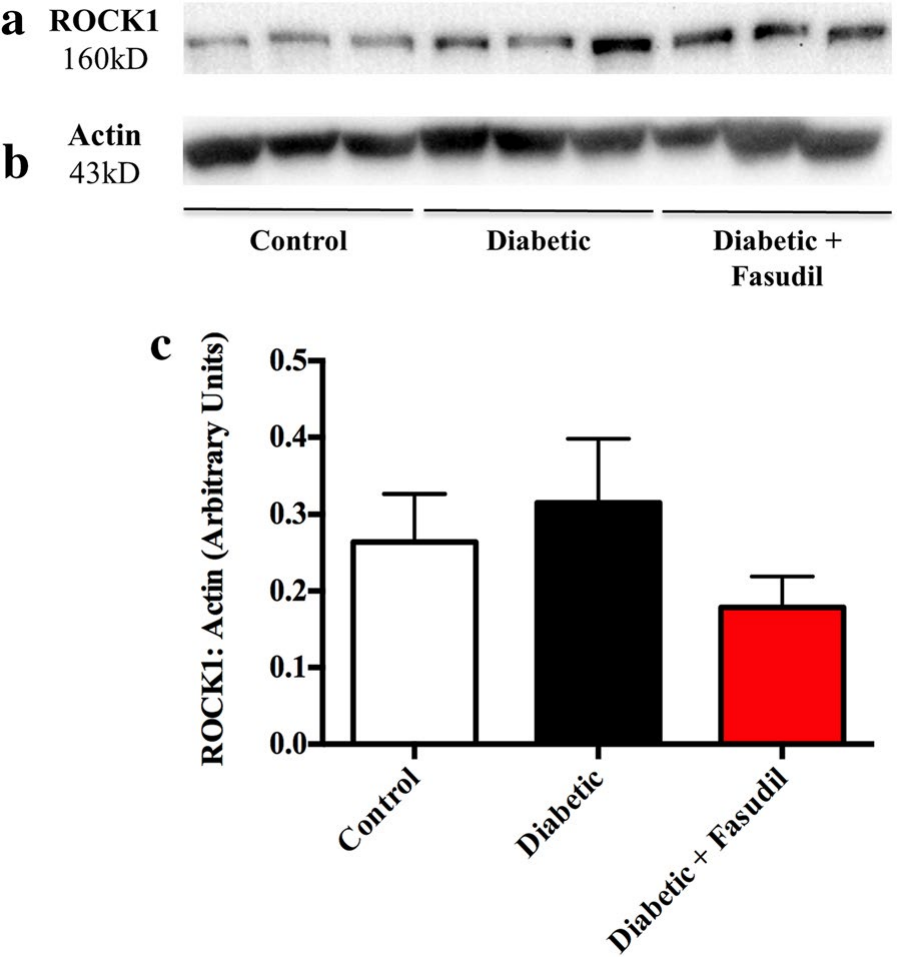
Rho kinase 1 (ROCK1) expression in the left ventricle. Representative images of western blot membranes for ROCK1 (**a**) and actin loading control (**b**) from control, diabetic and diabetic rats treated with fasudil (10 mg/kg/day). Compared to control rats, ROCK1 protein expression in the left ventricle of diabetic rats was similar. Fasudil treatment (10 mg/kg/day) in diabetic rats did not change ROCK1 expression in the left ventricle in comparison to the diabetic group. *Panel* **c** is the quantification of ROCK1 expression relative to actin. Data expressed as mean ± SEM. *N* = 6 per group.

**Figure 2:**
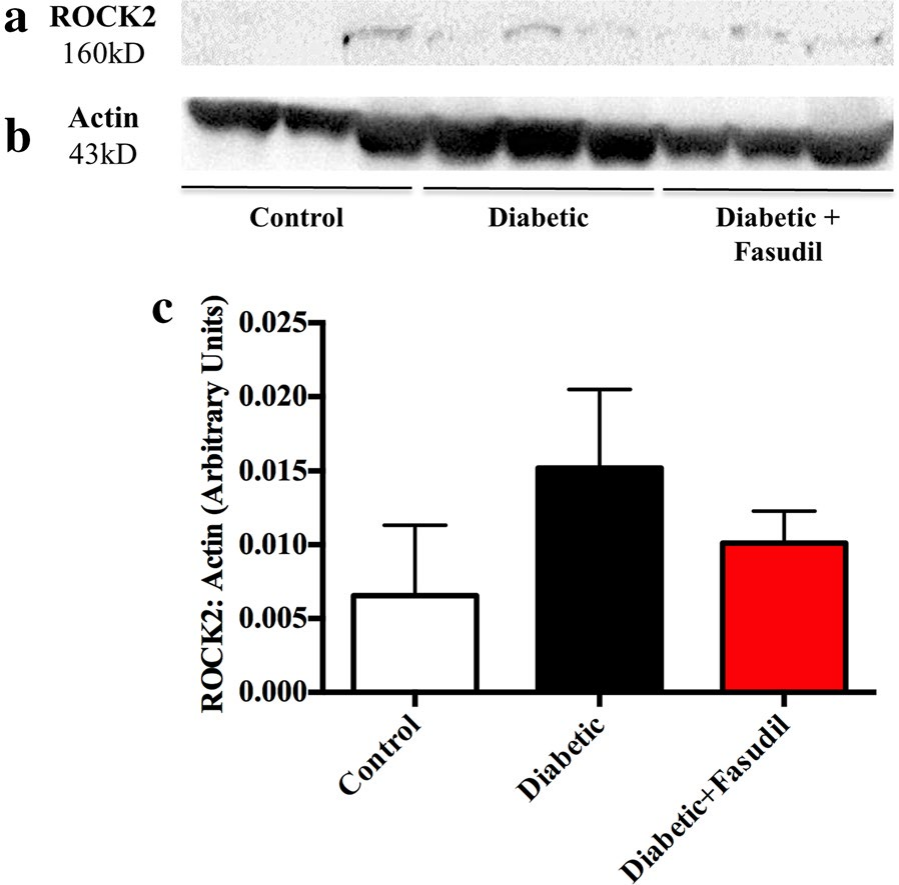
Rho kinase 2 (ROCK2) expression in the left ventricle. Representative images of western blot membranes for ROCK2 (**a**) and actin loading control (**b**) from control, diabetic and diabetic rats treated with fasudil (10 mg/kg/day). No significant changes were observed among the three experimental groups. *Panel* **c** is the quantification of ROCK2 expression relative to actin. Data expressed as mean ± SEM. *N* = 6 per group.

On average ROCK1 expression (Figure 1a) in the LV of diabetic group was ~19% higher than the control group (0.32 ± 0.08 vs. 0.26 ± 0.06 AU, NS, Figure 1c). Chronic treatment of diabetic rats with fasudil resulted in a ~48% lower ROCK1 expression in comparison to vehicle treated diabetic rats (0.18 ± 0.04 vs. 0.32 ± 0.08 AU, NS, Figure 1c). LV ROCK2 expression (Figure 2a) was elevated ~2.5-fold in diabetic rats, although this did not reach statistical significance (0.015 ± 0.005 vs. 0.006 ± 0.005 AU, Figure 2c). Two weeks of fasudil tended to reduce ROCK2 expression in the diabetic heart (0.015 ± 0.005 vs. 0.010 ± 0.002 AU, NS, Figure 2c). Thus, ROCK expression was not consistently increased in early diabetes as has been reported in advanced states of diabetes [35].

Three weeks of STZ-induced diabetes in rats resulted in a ~23% lower MyBP-C phosphorylation compared to control rats, which was partially attenuated by fasudil (Additional file 1: Figure  S2c). MLC-2 exhibited a small increase in phosphorylation in the LV of the diabetic group only (~17%, NS Additional file 1: Figure S2d). When compared to control rats, a weak trend for a higher TnI phosphorylation was found in diabetic rats (~30% higher, P = 0.1254, Additional file 1: Figure S2e), while TnI phosphorylation was similar to that of control group levels in fasudil treated diabetic rats (Additional file 1: Figure S2e). TnT phosphorylation was similar among the control, diabetic and diabetic fasudil-treated groups (Additional file 1: Figure S2f). Hence, the only notable trend was for diabetic rats to have greater phosphorylation of TnI, which was not found in fasudil treated diabetic rats.

### Actin–myosin cross-bridge dynamics: experiment 2

#### Myosin head proximity to actin thin filaments and interfilament spacing

**Figure 3:**
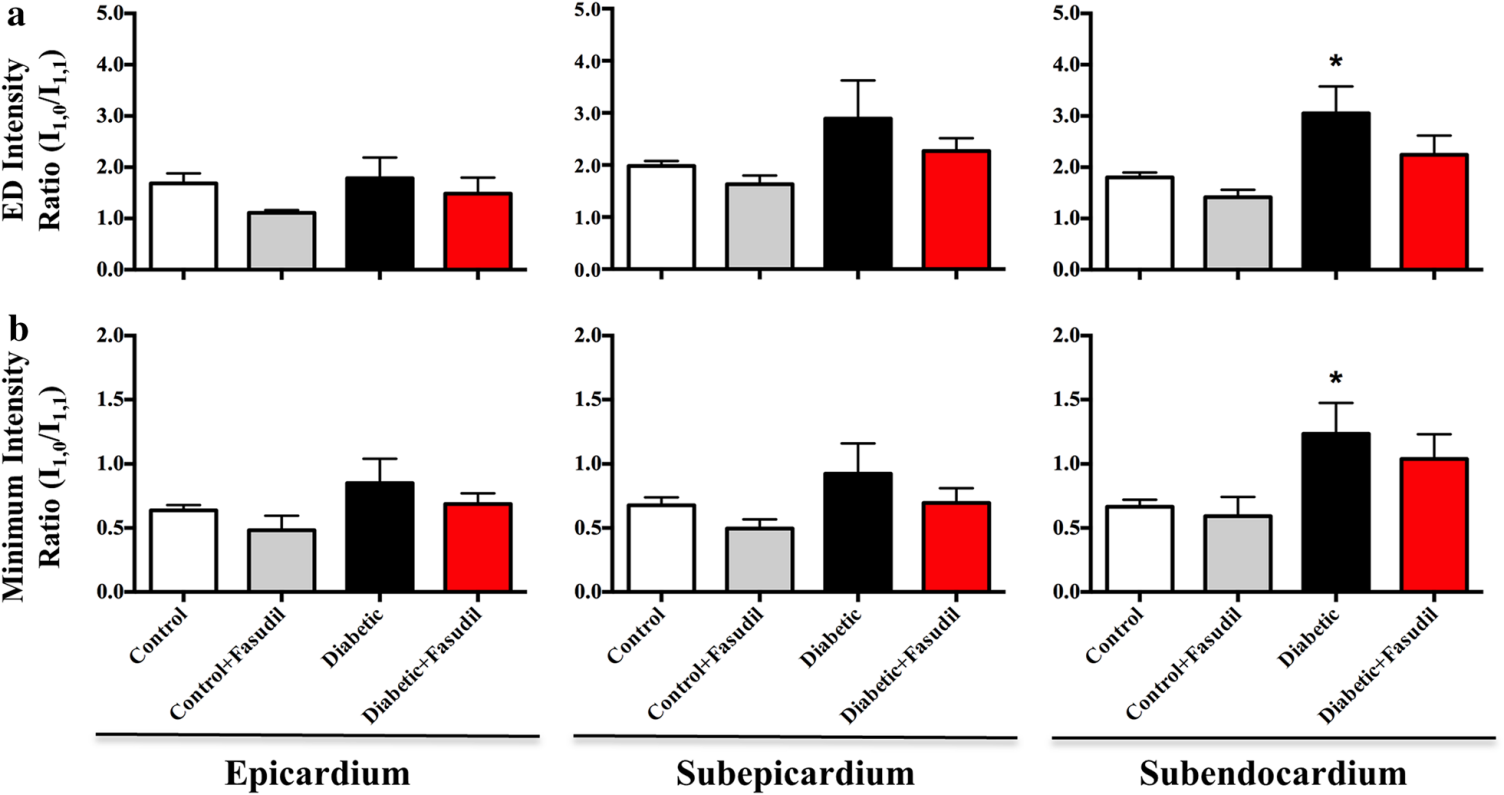
Myosin mass transfer changes across the cardiac cycle. Mean intensity ratio (I_1,0_/I_1,1_) at end diastole (ED, **a**) and systolic minimum (**b**) in the epicardial, subepicardial and subendocardial layers of the left ventricle. Intensity ratio did not significantly differ between vehicle-treated control and diabetic rats or fasudil-treated (10 mg/kg/day) control and diabetic rats in the epicardium or subepicardium at either time point. In the deep subendocardial layer, diabetic rats had a significantly elevated ED and systolic minimum intensity ratio (P < 0.05). Data expressed as mean ± SEM. *P < 0.05 vs. control in the same myocardial layer. *N* = 4–6 per group.

**Figure 4:**
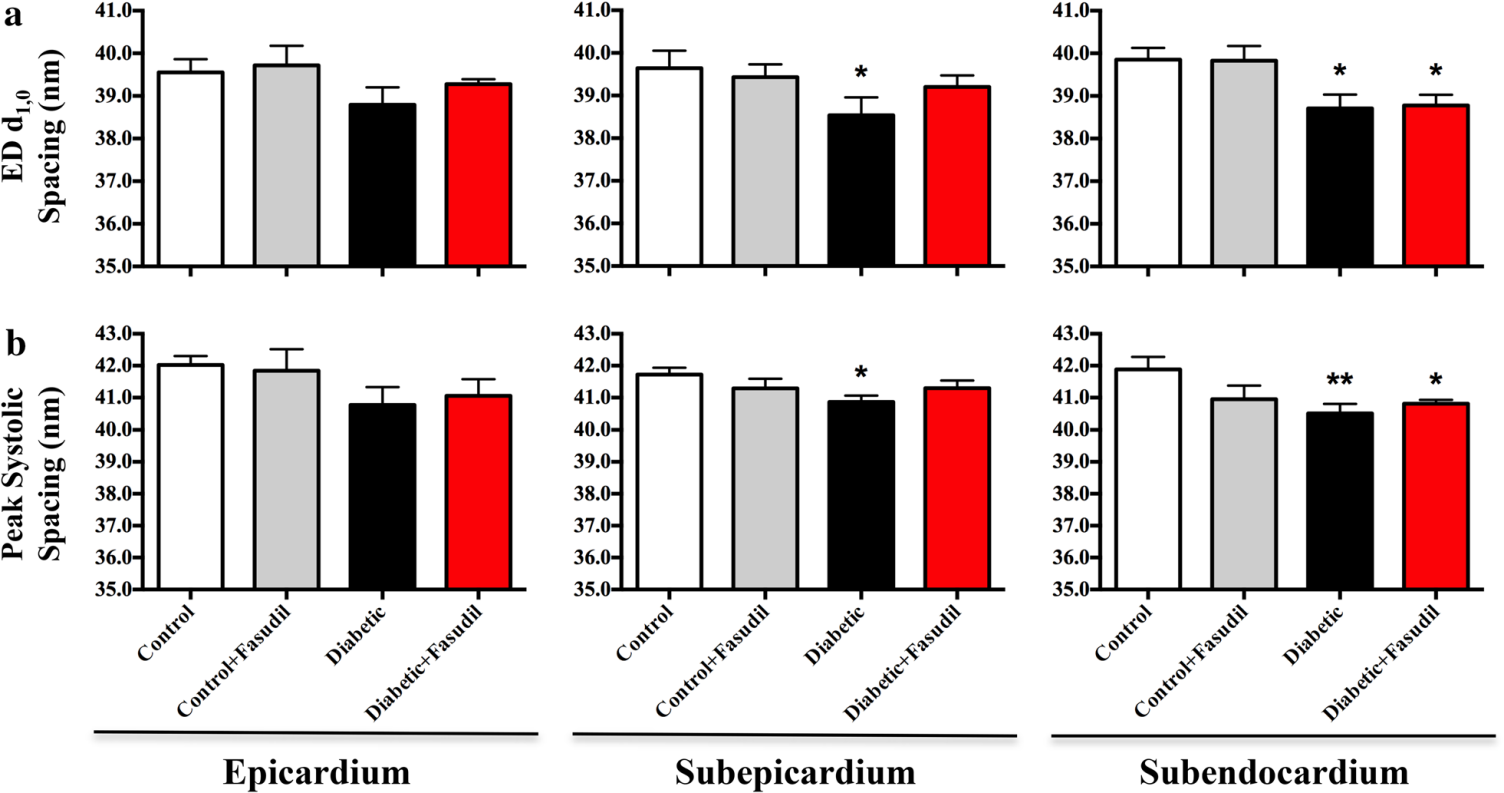
End diastolic (ED) interfilament (d_1,0_) spacing (**a**) and systolic interfilament spacing (**b**) from experiment 2. In comparison to control rats, ED and peak systolic d_1,0_ spacing in the was not significantly different in the epicardium between the groups. In the subepicardium, diabetic rats had a significantly smaller ED and systolic d_1,0_ spacing compared to control rats (P < 0.05). However, in the subendocardial layer, both the diabetic and diabetic rats treated with fasudil (10 mg/kg/day) had significantly reduced ED d_1,0_ spacing (P < 0.05) and systolic spacing (P < 0.01, P < 0.05, respectively) in comparison to control rats. Data is expressed as mean ± SEM. *P < 0.05 and **P < 0.01 vs. control in the same myocardial layer. *N* = 4–6 per group.

In control and fasudil-treated control rats, ED (Figure 3a) and minimum (representative of peak systolic CB attachments) intensity ratios (Figure 3b) did not significantly differ with respect to myocardial layer. Diabetic rats exhibited higher ED intensity ratios in all myocardial layers compared to control rats, and in contrast, intensity ratio was significantly higher in the subendocardium (P < 0.05, Figure 3a). Similarly, minimum intensity ratio in diabetic rats was higher in all myocardial layers (Figure 3b), increased with myocardial depth in comparison to control rats (subendocardium P < 0.05, Figure 3b). In diabetic rats treated with fasudil, neither ED or minimum intensity ratios differed significantly from the control group, but intensity ratio over the cardiac cycle was intermediate between control and saline treated diabetic groups. Elevated intensity ratios in diabetic rats in general were paralleled by significantly smaller ED and systolic d_1,0_ spacings (P < 0.05, Figure 4). Fasudil treatment resulted in intermediate myosin spacings with the greater difference from the control group in the subendocardium.

#### Absolute myosin mass transfer

**Figure 5:**
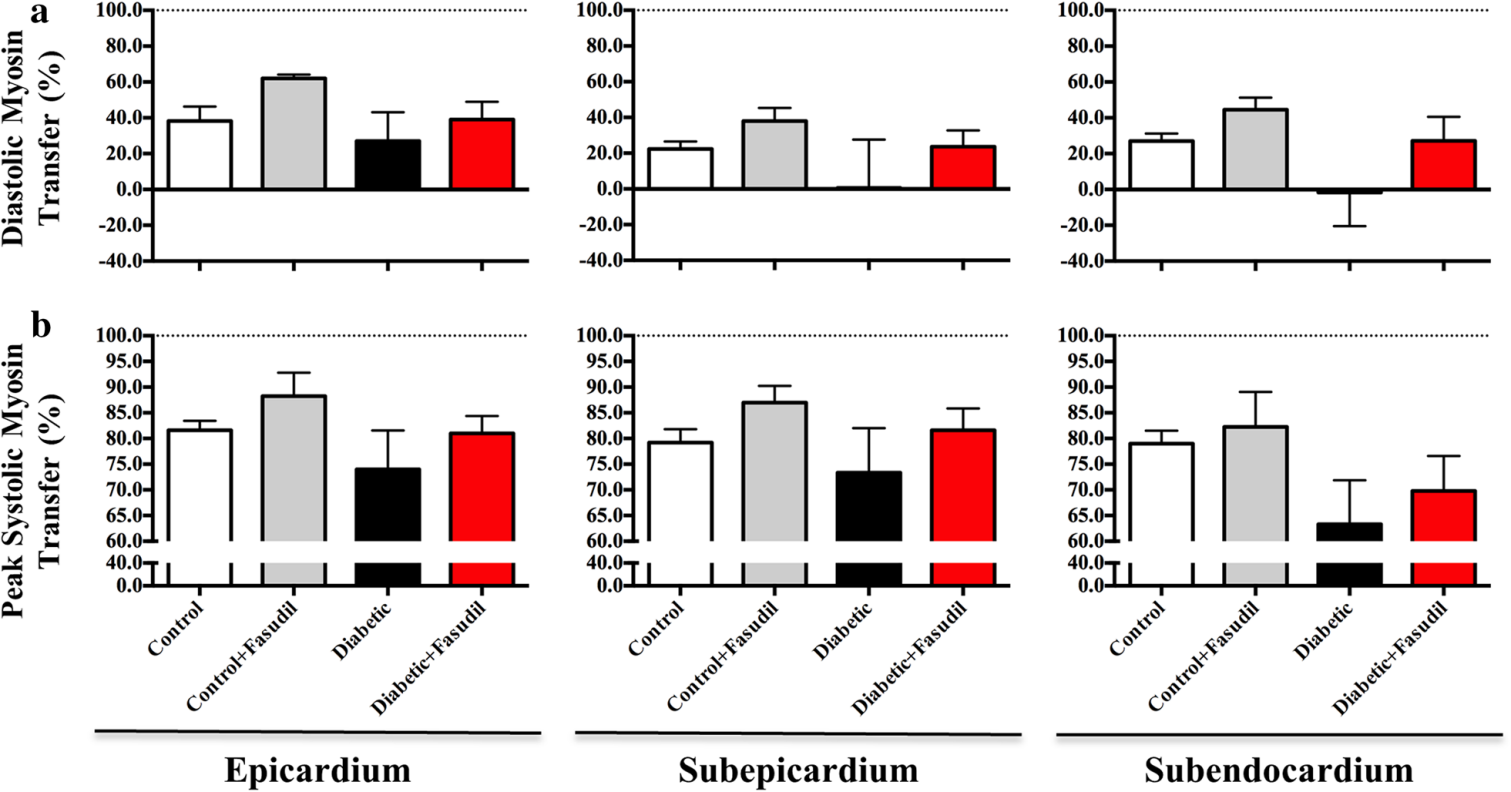
Diastolic and peak systolic myosin mass transfer. Diastolic (**a**) and peak systolic (**b**) myosin mass transfer in the epicardium, subepicardium and subendocardium of the left ventricle. Diastolic myosin mass transfer did not significantly differ between vehicle-treated control and diabetic rats or fasudil-treated (10 mg/kg/day) control and diabetic rats in the epicardial layer. In the subepicardial and subendocardial layers, no significant changes were observed in diastolic myosin mass transfer in diabetic rats compared to controls, or to fasudil-treated diabetic rats. Peak systolic myosin mass transfer was similar in all LV layers in diabetic rats compared to controls. Fasudil treatment of diabetic rats did not change subendocardial systolic myosin mass transfer. Data expressed as mean ± SEM. *N* = 4–6 per group.

In the two control groups, ~20–60% of myosin heads remained in close proximity to actin at ED (Figure 5a). In the epicardium, both diabetic and fasudil-treated diabetic groups were found to have on average comparable myosin mass transfer to actin to that of control group (27 and 39%, respectively) at ED relative to quiescent and rigor states of cardiac muscle [27] (Figure 5a). However, in the subepicardium and subendocardium, ED myosin mass transfer from the myosin thick filament backbone to actin was variable between individuals and on average zero in the diabetic group (Figure 5a). In contrast, the fasudil treated diabetic group maintained similar ED myosin mass transfer to the control group (Figure 5a), suggesting that ROCK may contribute to early impairment of diastolic CB dynamics in the diabetic heart.

Peak systolic myosin mass transfer was maintained at ~80–90% in both groups of control rats in all depths of the LV wall (Figure 5b). In the saline treated diabetic group, peak systolic myosin mass transfer was ~10 and ~8% lower in the epicardial and subepicardial layers, respectively in comparison to the control group (Figure 5b), while the diabetic group treated with fasudil exhibited similar mean peak systolic myosin mass transfer to that of the control group (Figure 5b). In the subendocardium, diabetic rats had a ~15% lower systolic myosin mass transfer compared to controls. Fasudil treatment of diabetic rats restored ~47% of the decrease in subendocardial systolic myosin mass transfer observed in diabetic vehicle treated group (Figure 5b).

**Figure 6:**
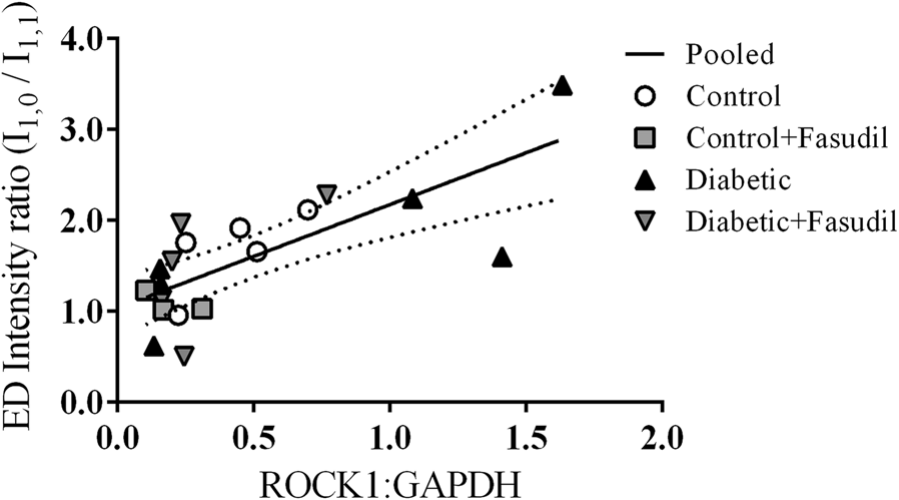
Correlation between ROCK1 protein expression and end diastolic myosin mass transfer in individual rats. ROCK1 expression in the left ventricle relative to the GADPH loading control is presented for the rats utilized in the SAXS experiment 2. End diastolic (ED) intensity ratio (I_1,0_/I_1,1_) is presented for the epicardial layer. A common linear regression fitted to pooled data for all rats is shown with 95% confidence intervals of the mean (*y* = 1.139*x* − 0.910, slope P < 0.0003, r^2^ = 0.554).

In experiment 2, similar to the cohort in experiment 1, ROCK1 expression tended to be higher in the diabetic group and was low in the fasudil treated groups (Additional file 1: Figure S4). Expression of the RhoA activator of ROCK did not differ significantly between groups. The large within group variability precluded significant difference in mean ROCK expression in the LV between groups. Nonetheless, there was a direct correlation between global ROCK1 expression and ED intensity ratio derived from the epicardium in individual rats (Figure 6). The fit of the regression for the diabetic group alone was similar to that of the common regression for all groups pooled (slope P < 0.056, r^2^ = 0.637 vs. pooled slope P < 0.0003, r^2^ = 0.554). This suggests that myosin head extension from the myosin filament is decreased as ROCK expression in the myocardium increases with diabetes progression.

#### Myosin head detachment in diastole

We used the rate of change in diffraction intensity ratio as an index of the rate of CB detachment from actin in early diastole. Additional file 1: Figure S6a shows the rate of change in intensity ratio over the cardiac cycle for individual rats. In the systolic phase, the rate of change is negative. From 30 to 40% of the cardiac cycle, the intensity ratio increases as CBs detach from actin (Additional file 1: Figure S6a). The maximum rate of intensity ratio increase occurred at 73–75% of the cardiac cycle in control rats treated with fasudil and in both groups of diabetic rats, which was significantly later than in control rats (63 ± 2.0%, P < 0.05, Additional file 1: Figure S6b). Thus, ROCK inhibition did not increase the rate of CB detachment.

#### Myosin head disposition in relation to global left ventricular function

ED intensity ratio in the epicardium was positively correlated with the minimum rate of LV pressure decay (global LV relaxation) in individual diabetic vehicle treated rats (slope P < 0.001, r^2^ = 0.95, Additional file 1: Figure S7). An elevated ED intensity ratio in the epicardium appears to correlate with global diastolic dysfunction in diabetic rats as the rats found to have low dP/dt minimum also had elevated ED intensity ratios in all layers of the heart. No such correlation was observed in control (vehicle or fasudil treated) or fasudil treated diabetic groups.

## Discussion

In the present study, we have observed a possible role for the ROCK pathway in the development of LV contractile dysfunction in early DCM. Our data revealed that LV systolic and diastolic dysfunction were associated with a trend for elevated expression of cardiac ROCK1 and ROCK2 in vehicle-treated diabetic rats, as reported by others [24, 35]. Importantly, we have demonstrated that chronic ROCK inhibition with fasudil maintained basal CB dynamics in diabetic rats as evidenced by preserved diastolic myosin mass transfer in all fiber layers of the LV wall. Further, chronic ROCK inhibition also partially rescued the suppressed subendocardial peak systolic myosin mass transfer observed in vehicle-treated diabetic rats. Fasudil exerted these beneficial effects in diabetic animals without changes in blood glucose concentrations or blood pressure. This suggests that the effects of ROCK inhibition with fasudil were found to directly target the myocardium and not the vasculature at this dose.

The rat model of early diabetes used in our studies (3 weeks post STZ-induced diabetes) allowed for the examination of early functional changes to the myocardium, independent of overt structural remodeling that is generally present in the more advanced stages of DCM [30, 36]. We found that cardiomyocyte cross-sectional area (indicative of hypertrophy) and interstitial fibrosis scores did not differ among the groups. This is consistent with our previous findings [24], and that of others [23] using rat models of early diabetes. Thus, it is likely that the mild cardiac dysfunction observed in diabetic rats at this time-point arises from subcellular alterations to contractile apparatus within the cardiomyocyte due to ROCK activation.

### Regional differences in cross-bridge dynamics in relation to global cardiac function: the effects of fasudil

Our previous study has reported elevated ED and minimum intensity ratios with increasing myocardial depth in diabetic rats, indicating regional differences in CB dynamics and thus, non-uniform regulation of CB dynamics across the LV wall in the diabetic heart [8]. In the present study, we confirmed these findings with the observation that pronounced myosin head displacement from actin at ED and peak systole occurred in the subepicardial–subendocardial layers in diabetic rats. These data are supported by clinical evidence from tissue Doppler imaging of myocardial velocity and strain rate suggesting that longitudinal contractility of the heart by the subepicardial–subendocardial layers declines prior to that of epicardial and endocardial fibers of the heart in diabetic patients [37].

### Systolic function and force development

Our findings demonstrate that fasudil treatment of diabetic rats maintains myosin heads away from the myosin backbone during diastole similar to that of control rats, in all layers of the LV wall. This is an important finding as early studies conducted by Matsubara and colleagues [38] revealed that the number of myosin projections in close proximity to actin during diastole is directly related to the developed force of subsequent contractions. Therefore, it is conceivable that the improvements in systolic performance and force development of diabetic rats treated with fasudil is likely to be a result of increased myosin head proximity to actin thin filaments in diastole.

In principle, differences in the interfilament spacing (and thus sarcomere length) could potentially explain the differences in ED myosin mass shifts. Previous work conducted by our group shows a direct inverse correlation between interfilament spacing and the number of CBs that can form attachments in the next cardiac cycle [28]. In the current investigation, we have found that in the diabetic heart, ED interfilament spacing was significantly smaller, in all myocardial layers, but particularly in the fiber layers below the epicardial surface (Figure 6). Therefore, a smaller interfilament spacing coincided with increased diffraction intensity ratio and reduced myosin head extension. Fasudil treatment resulted on average in a myosin spacing similar to control rats in the superficial muscle layers, but an intermediate myosin spacing persisted in the subendocardium. Based on a growing body of evidence we considered that one possibility is that in diabetic rats treated with fasudil the increase in myosin head proximity to actin filaments is facilitated by changes in the phosphorylation state of the myosin accessory proteins involved in the regulation of myosin head extension.

### Diastolic function

ROCK activation has been implicated in the prolonged myocardial relaxation associated with DCM. Acute inhibition of ROCK with Y-27632 or H-1152 in an isolated heart preparation rapidly alleviates the prolonged rates of LV pressure decay in rats with advanced DCM, confirming that ROCK drives diastolic dysfunction by direct actions on the sarcomere [39]. However in the present study, we did not observe a significant improvement in active relaxation during diastole in diabetic rats treated with fasudil. This is in contrast to a similar study, where Guan et al. [15] reported a significant improvement in dP/dt_minimum_ after 4 weeks of an equivalent dose of fasudil treatment in rats with advanced DCM (8 weeks post diabetes induction). The exact reason for the discrepancy between these studies is uncertain. One possible explanation is that long-term treatment of diabetic rats with fasudil may be required to detect significant improvements in active diastolic relaxation rates. It should also be noted that in that study [15], measures of systemic blood pressure were not presented. As elevated blood pressure is a reported complication of advanced STZ-induced diabetes in rats [40], it is difficult to delineate if the observed improvements in active diastolic function in diabetic rats from the previous study were due to the antihypertensive effects of fasudil [41], reducing cardiac loading conditions or direct effects on the myocardium. However, a recent clinical study in a small cohort of T2DM patients with impaired LV relaxation has reported 2 weeks of twice daily fasudil treatment (30 mg twice per day, intravenous) significantly improved various diastolic parameters including E/A wave ratio, deceleration time, E/e′ and isovolumetric relaxation time [20], providing further support of a role for ROCK in the development of diastolic dysfunction in DCM. Therefore, the evidence available does suggest that ROCK inhibitors improve systolic function in early diabetes and diastolic function at least in advanced diabetes.

### Possible mechanisms for Rho kinase in cross-bridge dysregulation

The findings of the present study corroborate those from our previous study that showed small increases in expression of myocardial ROCK1 and ROCK2 [24]. Importantly, ROCK protein expression is shown to be directly related to the degree of CB dysfunction and the rate of LV pressure decline in diastole in this study. Further, chronic inhibition by fasudil at a dose of 10 mg/kg/day prevented any increase in myocardial ROCK1 and ROCK2 expression in diabetic rats. Similar findings were found in rats from both experimental cohorts (Additional file 1: Figure S4). Over the past decade, ROCK has been identified as an emerging therapeutic target, mainly due to a growing body of evidence implicating ROCK in various cardiovascular pathologies (reviewed in [42, 43]). Recently, it has even been shown that ROCK isoforms can be rapidly induced by oxidative stress and exhibit differential protein expression throughout the LV and right ventricle myocardium reflecting the severity of contractile dysfunction and regional differences in the hypertrophic state associated with pressure overload [44]. The role of ROCK in the regulation of actin–myosin interactions is pertinent to this study. It is well established in smooth muscle cells that ROCK regulates the phosphorylation state of MLC-2 through its interaction with myosin light chain phosphatase [45]. Moreover, in isolated cardiomyocytes it has been shown that ROCK can directly phosphorylate MyBP-C [21]. X-ray diffraction experiments conducted on isolated cardiac muscle have demonstrated that increased phosphorylation of either MLC-2 or MyBP-C results in radial displacement of myosin heads away from the myosin thick-filament backbone toward actin [46, 47]. Therefore, it is reasonable to expect that decreased phosphorylation of MLC-2 and or MyBP-C would lead to less myosin heads in the vicinity of actin in diastole. In our study, MLC-2 phosphorylation did not differ among the groups and the lower MyBP-C phosphorylation in the vehicle-treated diabetic group was not significant. In contrast, Chung and colleagues [48] found that myocardial MLC-2 phosphorylation was significantly reduced in the early diabetic heart with no change in MyBP-C phosphorylation. However, Korte et al. [49] have reported significant reductions in MyBP-C phosphorylation in the myocardium of diabetic swine. Moreover, deletion of MyBP-C or reduction in its phosphorylation in transgenic mouse models and patients carrying MyBP-C mutations causes diastolic dysfunction with impaired relaxation that appear to be primarily due to a slower CB detachment rate relative to attachment rate (reviewed by Tong et al. [50]). While diabetic rats treated with fasudil in this study had phosphorylation levels of MyBP-C that did not differ from the control group and a faster maximum rate of LV pressure decay than the vehicle-treated diabetic group the relaxation time constant was not shorter. Fasudil treatment did not influence the rate of increase in intensity ratio in the first period of diastole. Therefore, it is unlikely that following ROCK inhibition that the prolongation of active relaxation time can be attributed to an ATP dependent limitation on the rate of CB detachment, even though it has been suggested that ROCK activation reduces actomyosin ATPase activity [21]. Taken together the available evidence suggests that ROCK activation mediates the early origins of contractile dysfunction, by reducing myosin head extension from the myosin backbone and reducing the probability of strong CB formation during systole. However, a role for altered total phosphorylation activity of cardiac troponins or MyBP-C in mediating the effects of ROCK activation in early contractile dysfunction is not supported by our findings. This is not to say that the ROCK mediated reduction in MyBP-C phosphorylation activity does not become more pronounced in advanced diabetes, but rather other accessory proteins may be more important in modulating myosin head extension in early diabetes, such as titin.

### Limitations and future directions

In the present investigation we have presented data for ROCK1 and ROCK2 expression in the hearts of rats, however an important limitation of our study is we did not demonstrate specific activation of ROCK target proteins to confirm increased ROCK activity. Other studies have reported that phosphorylation of downstream targets of ROCK including myosin light chain phosphatase targeting subunit 1 (MYPT1), LIM kinase (LIMK) and the ezrin/radixin/moesin (ERM) complex are elevated in the hearts and aorta of rats with advanced DCM [15, 34, 35, 39, 51, 52], indicating increased activity of ROCK. Utilising whole-heart homogenates from the rats used in our studies of LV function we were not able to obtain clear western blot bands for LIMK or MYPT1 phosphoproteins to demonstrate elevated ROCK activity, in contrast to the previous studies that utilized aorta or isolated cardiomyocytes [34, 35, 52]. In addition, previous studies in general detected low absolute levels of protein in advanced DCM [39, 51], whereas this study focused on early diabetes where expression levels might be expected to be lower. Considering this limitation of the current study future studies might benefit from investigating ROCK activity in isolated cardiomyocytes derived from diabetic rats at this early stage of DCM.

Although synchrotron SAXS was sensitive enough to be able to detect regional difference in CB dynamics, we did not assess regional changes in sarcomeric protein phosphorylation in the present study. In the hearts of rats post myocardial infarction, a reduction in MLC-2 phosphorylation was shown to primarily occur in the endocardial myocytes, while no changes in MLC-2 phosphorylation were observed in the epicardium [53]. To the best of our knowledge, no such studies of regional changes in phosphorylation state have been conducted in the context of DCM. Future studies that examine regional changes in thick and thin filament accessory protein phosphorylation in parallel with regional SAXS recordings may further enhance our understanding of the pathogenesis of DCM.

The giant sarcomeric protein titin, (among various other functions) is an important regulator of CB kinetics and dynamics in the myocardium by regulating cardiomyocyte passive tension. Titin stiffness is primarily modulated via changes in titin isoform composition and or phosphorylation state. In rodent models of advanced T1DM and T2DM, titin isoform shifts from the stiffer N2B to the more elastic N2BA [54] and hypophosphorylation of the N2B isoform [55] are associated with diastolic dysfunction. Studies in isolated rat ventricular trabeculae have suggested that titin regulates actin–myosin interaction by interfilament lattice spacing modulation [56]. However, the role of interfilament spacing in the modulation of CB formation in length dependent activation remains controversial. Interestingly, Hanft et al. [57] have shown that myosin head flexibility and extension towards to actin increases when titin is slackened. The significantly smaller myosin interfilament spacing in the vehicle-treated diabetic group relative to the control group predicts that sarcomere length would be longer throughout the cardiac cycle (Figure 4), and therefore more taut, albeit within the normal sarcomere operating range. Whether ROCK activation influences myosin head extension and CB dynamics in vivo through changes in titin isoform and or phosphorylation state remains to be investigated.

## Conclusion

In summary, we have confirmed that impairment in regional CB dynamics contribute to a reduction in developed force and LV contractile dysfunction in early DCM. Furthermore, our current work suggests that increased ROCK expression and activation is involved in the development of DCM as chronic inhibition of ROCK with fasudil preserved diastolic myosin mass transfer and prevented the decline in LV systolic function and force development. These findings appear to be largely independent of changes in TnI, TnT, MLC-2 or MyBP-C protein phosphorylation. Nevertheless, these data suggest that ROCK is an important and promising therapeutic target for the treatment and management of DCM.

## Additional file

Additional file 1: Supplementary material including interstitial fibrosis scores and cardiomyocyte cross-sectional areas, and Western blot data for Rho-kinase and RhoA from experiment 2 are presented. Also included are relative phosphorylation states of myofilament proteins, rate of change of myosin mass transfer and the correlation between dP/dt_minimum_ and ED intensity ratio.

## Abbreviations

ATP: adenosine triphosphate
BW: body weight
CB: cross-bridge
DCM: diabetic cardiomyopathy
dP/dt: rate of pressure change
ED: end diastole
EDPVR: end diastolic pressure volume relationship
EDV: end diastolic volume
ERM: ezrin/radixin/moesin
ESPVR: end systolic pressure volume relationship
H&E: haematoxylin and eosin
IVC: inferior vena cava
KCl: potassium chloride
LIMK: LIM kinase
LV: left ventricle
MLC-2: myosin light chain-2
MyBP-C: myosin binding protein-C
MYPT1: myosin light chain phosphatase targeting subunit 1
PRSW: preload recruitable stroke work
PV: pressure–volume
ROCK: Rho kinase
SAXS: small angle X-ray scattering
STZ: streptozotocin
T1DM: type 1 diabetes mellitus
T2DM: type 2 diabetes mellitus
TnI: troponin I
TnT: troponin T
