# Supplementary material for: Chronic Rho-kinase inhibition improves left ventricular contractile dysfunction in early type-1 diabetes by increasing myosin cross-bridge extension
Source: Cardiovasc Diabetol. 2015 Jul 22;14:92. doi: 10.1186/s12933-015-0256-6 (PMC4509700; doi:10.1186/s12933-015-0256-6)
Supplement: Supplementary file 1 — Supplementary material including interstitial fibrosis scores and cardiomyocyte cross-sectional areas, and Western blot data for Rho-kinase and RhoA from experiment 2 are presented. Also included are relative phosphorylation states of myofilament proteins, rate of change of myosin mass transfer and the correlation between dP/dtminimum and ED intensity ratio. [file 12933_2015_256_MOESM1_ESM.docx]

**Supplementary Material**

**Chronic Rho-Kinase Inhibition Improves Left Ventricular Contractile Dysfunction in Early Type-1 Diabetes by Increasing Myosin Cross-bridge Extension**

Mark T Waddingham^1^: m.waddingham@student.unimelb.edu.au

Amanda J Edgley^1,2^: aedgley@unimelb.edu.au

Alberto Astolfo^3,4^: a.astolfo@ucl.ac.uk

Tadakatsu Inagaki^5^: inagaki.tadakatsu.ri@ncvc.go.jp

Yutaka Fujii^5^: yfujii@ncvc.go.jp

Cheng-Kun Du^5^: duchk@ri.ncvc.co.jp

Dong-Yun Zhan^5^: dyzhan@ri.ncvc.co.jp

Hirotsugu Tsuchimochi^5^: tsuchimo@ri.ncvc.go.jp

Naoto Yagi^6^: yagi@spring8.or.jp

Darren J Kelly^1^: dkelly@medstv.unimelb.edu.au

Mikiyasu Shirai^5^: shirai@ri.ncvc.go.jp

James T Pearson^2,3,7*^: james.pearson@monash.edu

^1^Department of Medicine, St. Vincent’s Hospital, University of Melbourne, Melbourne, Victoria, Australia

^2^Department of Physiology, Monash University, Clayton, Victoria, Australia

^3^Australian Synchrotron, Clayton, Victoria, Australia

^4^Department of Medical Physics and Bioengineering, University College of London, London, England

^5^Department of Cardiac Physiology, National Cerebral and Cardiovascular Center Research Institute, Suita, Osaka, Japan

^6^Japan Synchrotron Radiation Research Institute, Harima, Hyogo, Japan

^7^Monash Biomedical Imaging Facility, Monash University, Clayton, Victoria, Australia

**^*^Corresponding Author**

**Supplementary Table S1** General characteristics of rats from experiment 1.

|  | **Control** | **Diabetic** | **Diabetic + Fasudil** |
| --- | --- | --- | --- |
| ***N*** | 8 | 9 | 12 |
| **Body Weight (g)** | 430 ± 12.2 | **331 ± 10.2^##^** | **321 ± 8.9^##^** |
| **Fasted Blood Glucose (mmol/L)** | 8.1 ± 0.2 | **26.4 ± 0.8^##^** | **28.7 ± 1.2^##^** |
| **Mean Arterial Pressure (mmHg)** | 113.0 ± 13.2 | 108.0 ± 13.2 | 116.0 ± 8.2 |
| **Left Venticle Weight (g)** | 0.8 ± 0.03 | **0.65 ± 0.03^#^** | **0.59 ± 0.02^##^** |
| **Left Ventricle Weight : Body Weight (mg/ g)** | 3.3 ± 0.2 | 4.1 ± 0.13 | 4.0 ± 0.13 |

Data expressed as Mean ± SEM. ^#^P<0.001 and ^##^P<0.0001 vs. Control rats.


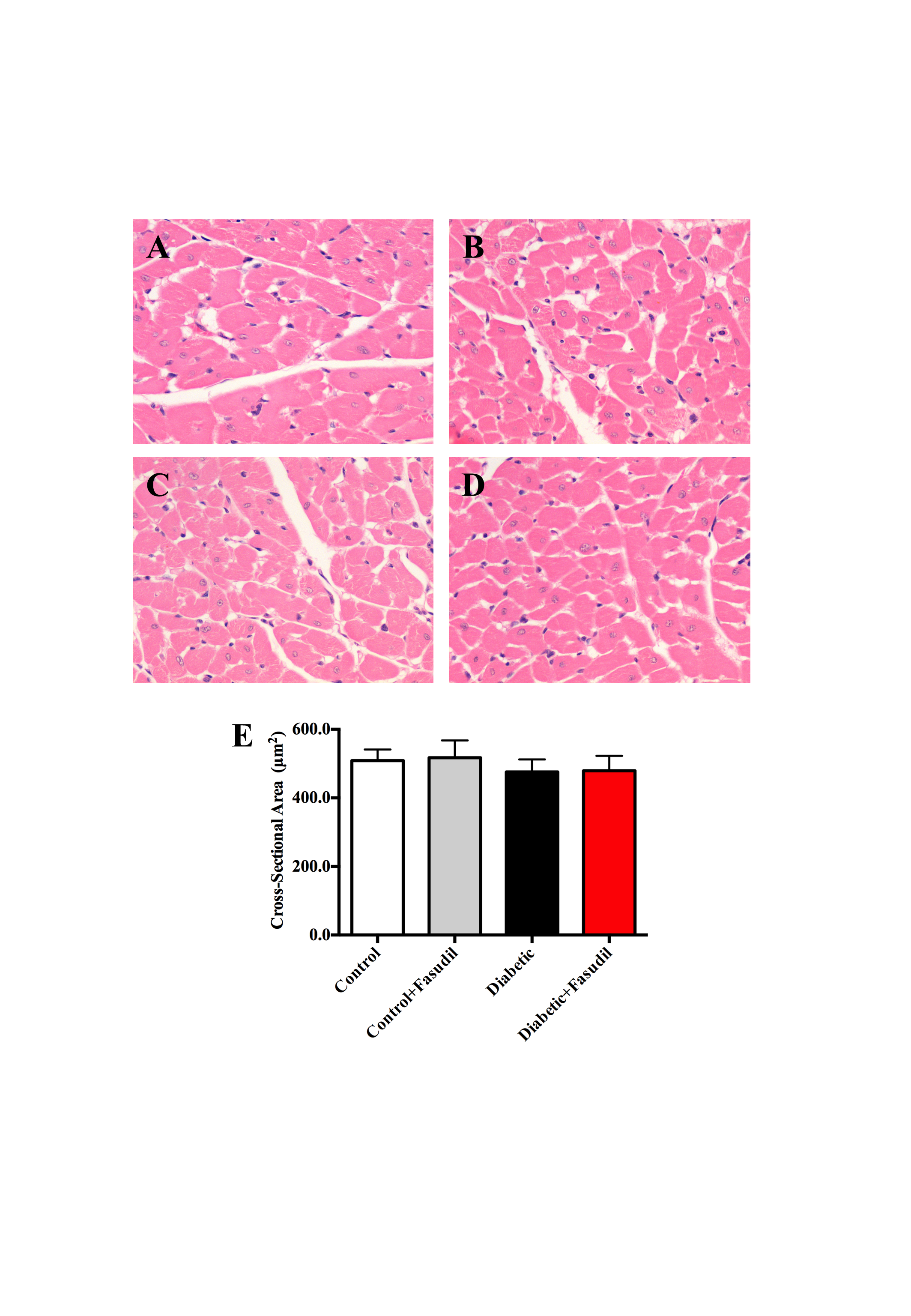


**Supplementary Fig. S1** Cardiomyocyte cross-sectional area. Representative images of heart sections from Experiment 2 stained with haematoxylin and eosin to assess cardiomyocyte cross-sectional area. Three weeks of STZ diabetes in rats (C) did not result in a significant change in cardiomyocyte cross-sectional area in comparison to control rats (A). Fasudil treatment (10mg/kg/day) did not affect cardiomyocyte cross-sectional area in control (B) or diabetic (D) rats. E is the quantification of cardiomyocyte cross-sectional area. Original magnification is x400. Data expressed as mean ± SEM. n=3-6 per group. Similar findings were also found for Experiment 1.

**
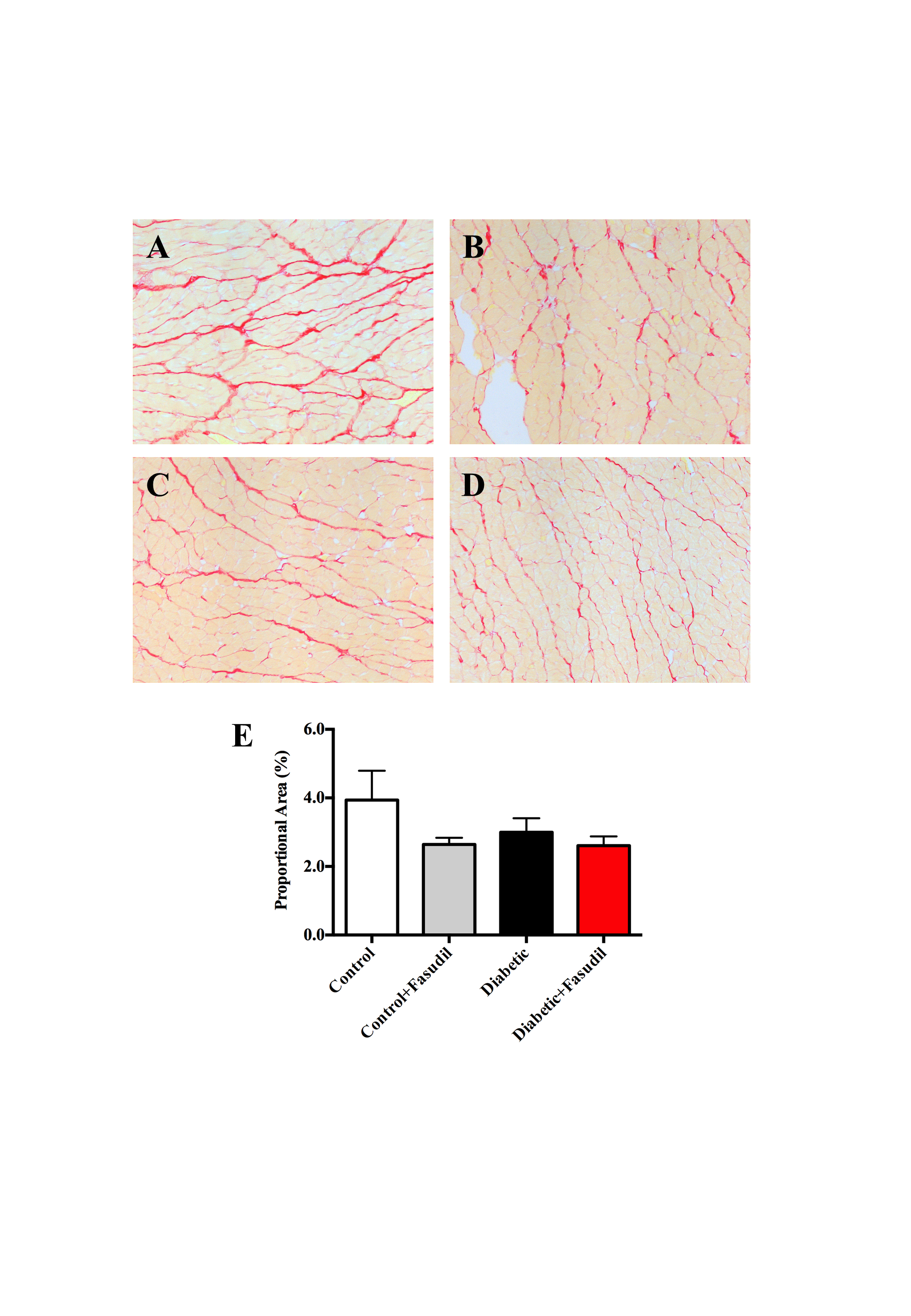
**

**Supplementary Fig. S2** Left ventricle interstitial fibrosis. Representative images of heart sections from Experiment 2 stained with picrosirius red to demonstrate LV interstitial fibrosis (red staining). Compared to control rats (A), three weeks of STZ diabetes in rats (C) did not affect LV interstitial fibrosis. Fasudil treatment (10mg/kg/day) did not significantly affect LV interstitial fibrosis score in either control (B) or diabetic (D) rats. E is quantification of the proportional area of red staining in each section. Original magnification is x200. Data expressed as mean ± SEM. n=3-7 per group. Similar findings were also found for Experiment 1.


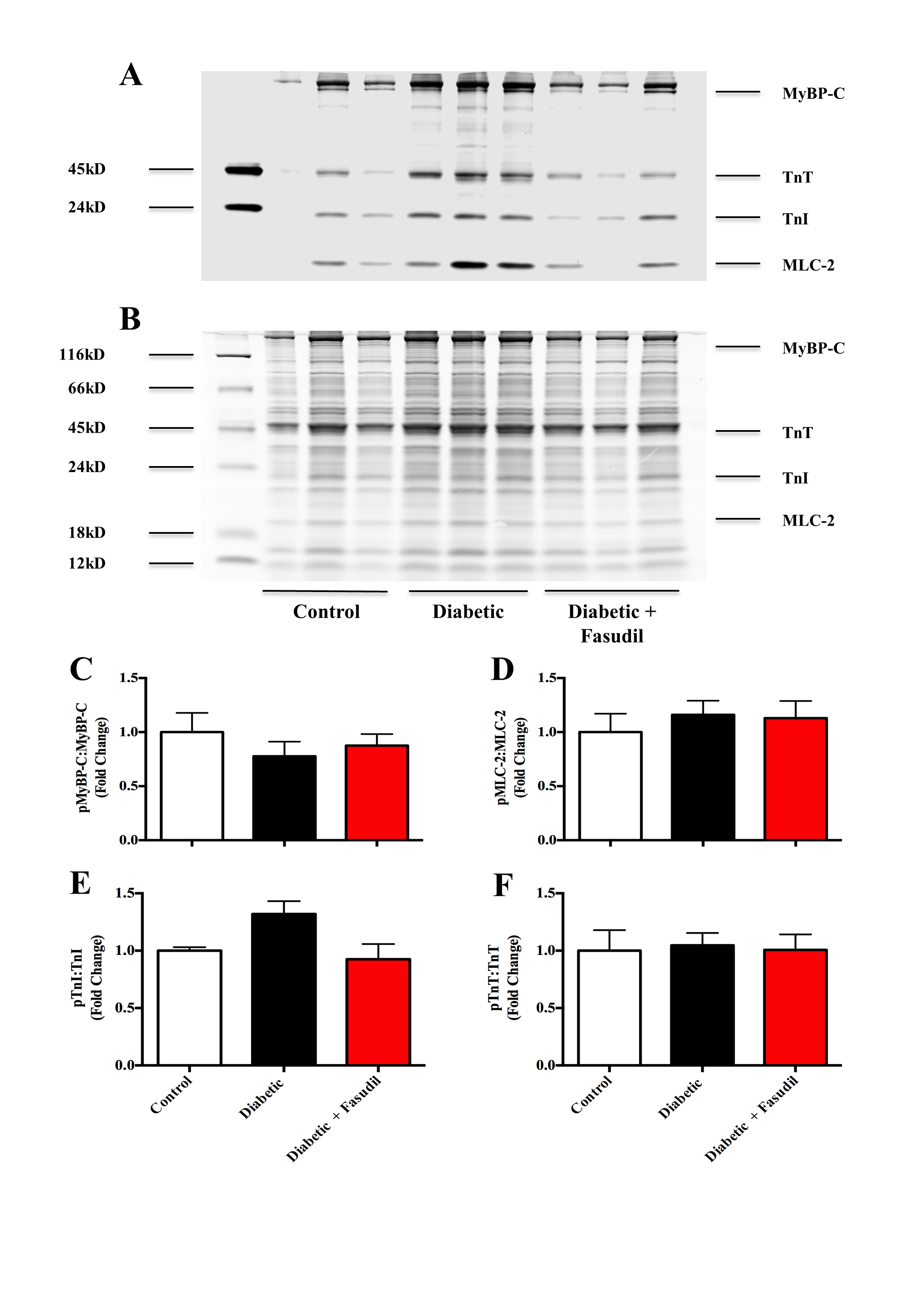


**Supplementary Fig. S3** Relative phosphorylation of myofilament proteins. Representative images of polyacrylamide gels stained with ProQ Diamond (A) for phosphoproteins and SYPRO Ruby for total protein (B) in Control, Diabetic and Diabetic + Fasudil (10mg/kg/day) rats. Panels C-F depict the quantification of the relative phosphorylation of MyBP-C (C), MLC-2 (D), TnI (E) and TnT (F). Data expressed as mean ± SEM. n=4-6 group.


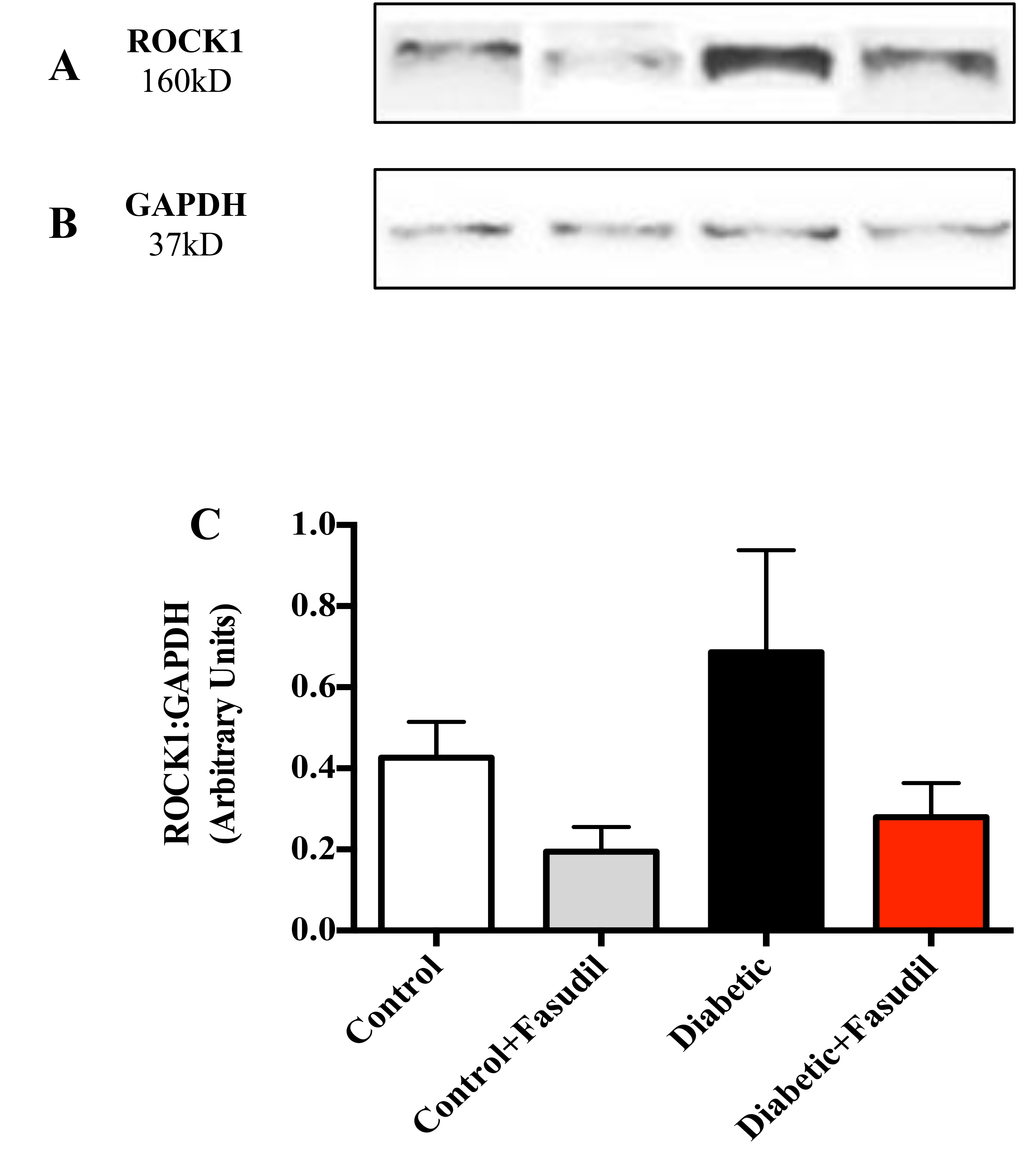


**Supplementary Fig. S4** Rho Kinase 1 (ROCK1) expression in the left ventricle in experiment 2. Representative images of western blot bands for ROCK1 (A) and GAPDH loading control (B) from control and diabetic rats treated with vehicle or fasudil (10mg/kg/day). Compared to control rats, there was a non-significant increase in ROCK1 expression in the left ventricle of diabetic rats. Fasudil treatment (10mg/kg/day) in control and diabetic rats resulted in a decrease of ROCK1 expression in the left ventricle when compared to their respective untreated groups. Panel C is the quantification of ROCK1 expression relative to GAPDH loading control. Data expressed as mean ± SEM. *n* = 3-6 per group.

**
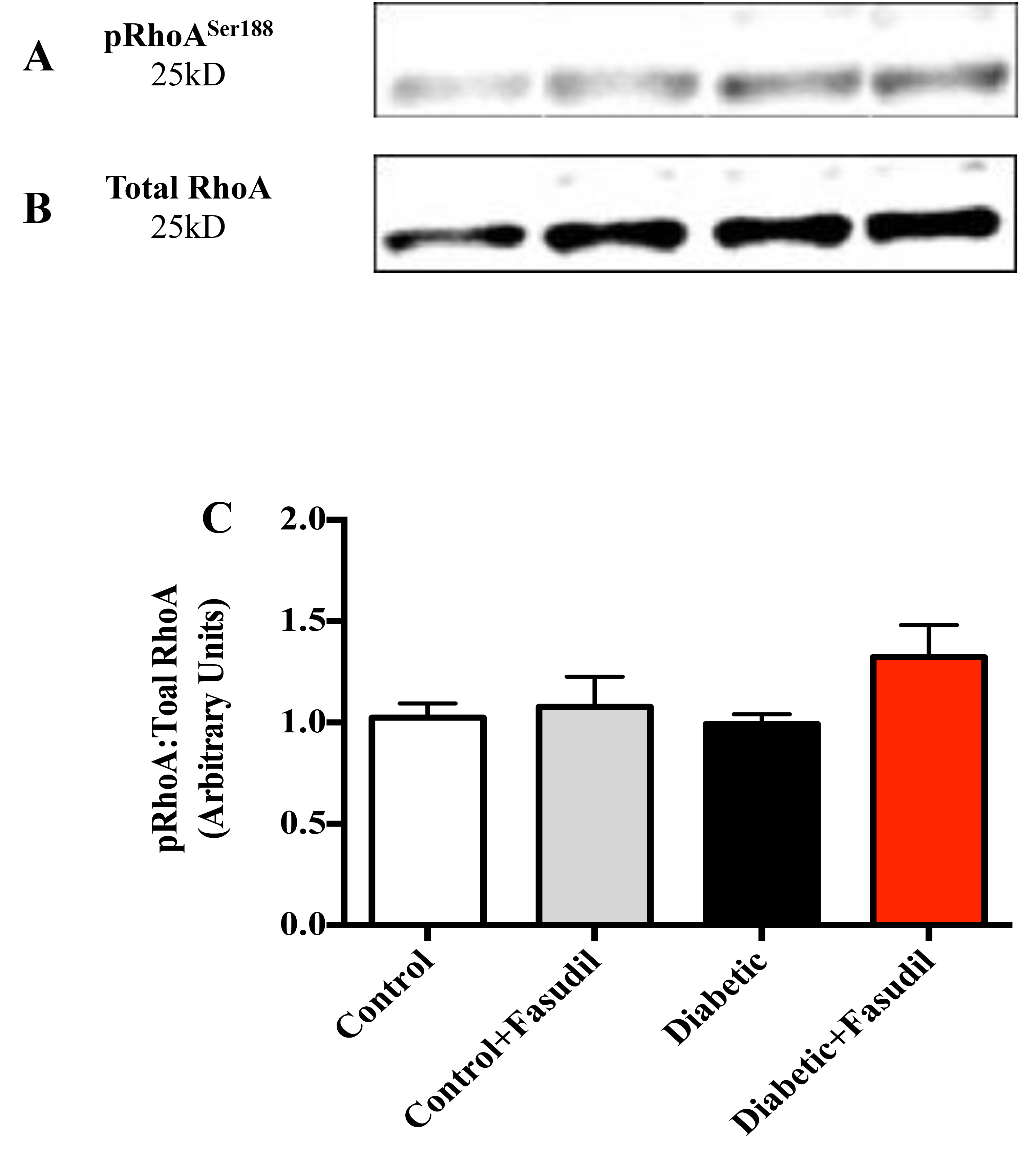
**

**Supplementary Fig. S5** Relative phosphorylation of RhoA in the left ventricle in experiment 2. Representative bands of western blots probed for phosphorylated RhoA at Ser188 (A) and total RhoA (B). Relative phosphorylation of RhoA did not significantly differ between control and diabetic rats treated with saline or fasudil (10mg/kg/day). Panel C is quantification of the relative phosphorylation of RhoA. Data expressed as mean ± SEM. *n* = 3-6 per group.





**Supplementary Fig. S6** Rate of change in myosin mass transfer in relation the cardiac cycle in experiment 2. Representative line profiles (A) of the rate of change in intensity ratio from control and diabetic rats treated with vehicle or fasudil over the cardiac cycle (represented as a percentage). In comparison to the control group, both diabetic groups and control rats treated with fasudil exhibited a significantly (*P*<0.05) prolonged time to maximum rate of change in intensity ratio and therefore a slower rate of myosin head detachment (B). Data expressed as mean ± SEM. *P<0.05 vs. Control rats. *n* = 4-7 per group.


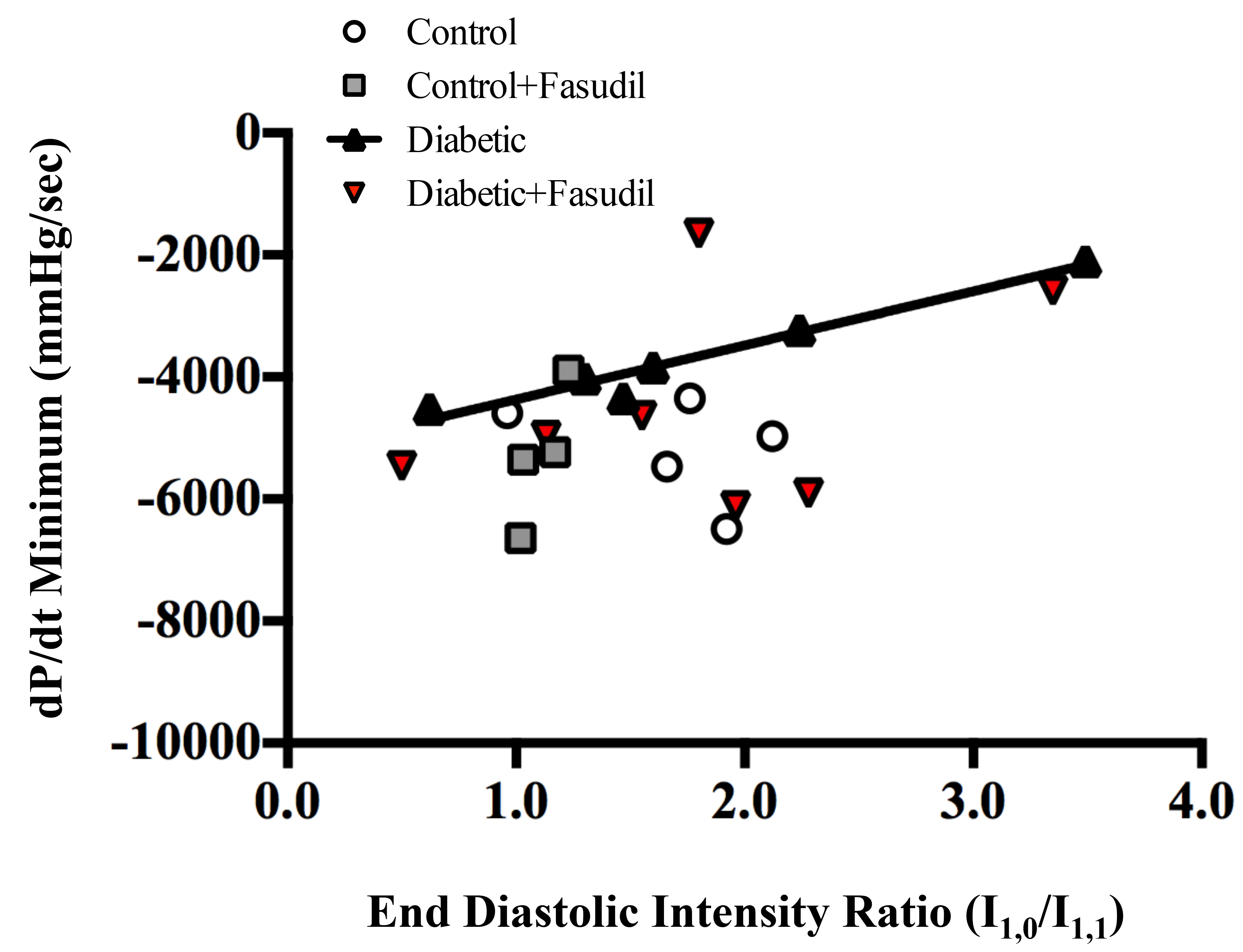


**Supplementary Fig. 7** Diastolic relaxation rate (dP/dt Minimum) in relation to end diastolic intensity ratio in experiment 2. The slope for control rats, control+fasudil rats (10mg/kg/day) and diabetic+fasudi rats (10mg/kg/day) did not significantly differ from zero. In diabetic rats, there was a significant increase in the slope from zero (P<0.001 r^2^= 0.95). Data presented from the epicardial layer. *n* = 4-6 per group.
